# Longer-term chiropractic care outcomes for US active-duty military personnel with low back pain: secondary analysis of a pragmatic clinical trial

**DOI:** 10.1186/s12906-026-05347-w

**Published:** 2026-04-07

**Authors:** Zacariah K. Shannon, Cynthia R. Long, Robert D. Vining, Jacob McCarey, Joan A. Walter, Ian D. Coulter, Christine M. Goertz

**Affiliations:** 1 Palmer College of Chiropractic, Davenport, IA USA; 2 Independent researcher, Silver Spring, MD, , USA; 3 RAND Corporation, Santa Monica, CA USA; 4 Duke University School of Medicine, Durham, NC USA

**Keywords:** Pragmatic clinical trial, Chiropractic, Pain management, Back pain, Complementary therapies

## Abstract

**Background:**

Few trials have directly evaluated outcomes of adding chiropractic care to usual medical care, and prior work has been limited to short-term follow-up. The objective of this work is to address these gaps by evaluating outcomes from the addition of chiropractic care to usual medical care in active-duty U.S. military members with low back pain (LBP) over 52 weeks.

**Methods:**

A multi-site, pragmatic clinical trial allocated 750 U.S. active-duty military personnel with LBP to usual medical care plus chiropractic care or usual medical care alone with the primary endpoint at 12 weeks. The final 154 participants enrolled in the study were asked to provide longer-term follow-up data. We used inverse probability weighting to account for missing outcome data and analyzed data using linear mixed-effects regression models over all follow-up time points. We report between-group mean differences at 12 and 52 weeks adjusted by baseline age, sex, site, pain duration, worst pain intensity, and interaction terms for site differences by group over time. Primary outcomes were disability (Roland-Morris Disability Questionnaire: RMDQ) and pain intensity (NRS). Secondary outcomes were PROMIS-29: pain interference, physical function, fatigue, sleep disturbance, and social role.

**Results:**

A total of 144 participants provided outcome data after 12 weeks. Overall, the mean participant age was 33 years, 113 (78%) were male, 54 (38%) were non-white race, 18 (13%) were Hispanic or Latino ethnicity, and 71 (49%) had chronic LBP. At 12 weeks, moderate between-group difference in RMDQ (2.2 points, 95% CI 0.4 to 4.0) favored chiropractic care with small difference in NRS (0.6, -0.1 to 1.2). At 52 weeks, RMDQ difference was small (1.7, -0.2 to 3.7) and NRS difference was negligible (0.2, -0.5 to 1.0). Improvement in PROMIS domains varied, with many diminishing over the course of follow-up; sleep disturbance demonstrated the greatest longer-term difference in improvement at 52 weeks (3.6, 0.5 to 6.7).

**Conclusions:**

Adding chiropractic care to usual medical care for active-duty U.S. military members with low back pain resulted in a small difference in pain-related disability and a meaningful difference in sleep disturbance but not pain intensity at 52 weeks. Differences in pain interference, physical function, and social role estimates also favored the addition of chiropractic care over the longer-term follow-up.

**Trial Registration:**

This trial was first posted for registration on clinicaltrials.gov (NCT01692275) on 09/06/12.

## Introduction

Low back pain (LBP) results in immense societal costs; it is a leading cause of global disability [1] and U.S. healthcare spending [2]. LBP is highly prevalent among people with different sociodemographic characteristics, including U.S. military members, for whom LBP has been the leading cause of medical visits for more than 10 years [3–13]. This can likely be attributed to overuse injuries and the high physical burden of military training and operations [14].

Treatment guidelines for low back pain in U.S. military members [15] are similar to those of the general population [16], emphasizing noninvasive and nonpharmacological interventions as first-line therapies. Recommended interventions include exercise, heat, massage, spinal manipulation, other mind-body therapies, such as mindfulness-based stress reduction, tai chi, yoga, and progressive relaxation [16]. These interventions are delivered by a variety of practitioners, including medical physicians, physical therapists, and chiropractors, with guidelines recommending integrative, team-based care [15].

Though team-based care is recommended, effects attributable to combining care from multiple providers are not well understood. Few studies have evaluated both short term and longer term effects of adding chiropractic care to usual medical care [17]. The longer-term benefit of spinal manipulation, the signature intervention of chiropractic care, compared to other recommended therapies has previously been reported to diminish by 52 weeks [18]. However, chiropractic care is typically delivered as a multimodal combination of interventions including patient education and self-management strategies, active interventions such as exercise, and passive interventions such as spinal manipulation and other manual therapies [19–21]. Evaluating longer-term outcomes of adding chiropractic care to usual medical care in a pragmatic setting is required to better inform outcome expectations for U.S. military personnel with LBP.

A pragmatic clinical trial addressed this gap by evaluating the effect of adding chiropractic care to usual medical care for U.S. military members with low back pain. Study authors found a moderate difference in improvement in pain-related disability at the end of 6-weeks of care [22, 23]. The original trial design included follow-up 12 weeks after baseline [22] predicated on anticipated combat deployment of sample participants. Because a 12-week follow-up period limits the ability to draw conclusions about longer-term effects, additional follow-up over 52 weeks was added during later stages of recruitment. The objective of this work is to evaluate and report pain-related and quality of life outcomes of chiropractic care added to usual medical care for active-duty U.S. military members with low back pain using data from a subsample of this pragmatic clinical trial.

## Methods

### Trial design, setting, and participants

The trial was prospectively registered (ClinicalTrials.gov identifier: NCT01692275) and the protocol was previously published [22]. The trial was a multi-site pragmatic clinical trial comparing the effects of usual medical care plus chiropractic care vs. usual medical care alone for U.S. active-duty military personnel with LBP. The trial recruited 750 active-duty military personnel, including 250 at each of 3 different military treatment facilities: Walter Reed in Bethesda, Maryland; Naval Hospital in Pensacola, Florida; and the Naval Medical Center in San Diego, California. The trial protocol included a follow-up duration of 12 weeks after enrollment, of which the primary [23] and secondary [24] results have been previously published. The final 154 of the 750 enrolled participants consented to provide follow-up data over 52 weeks after enrollment. This included only participants from the Bethesda and Pensacola sites who enrolled between October 2014 and November 2015, the end of enrollment.

The trial protocol and longer-term follow-up amendment were approved by institutional review boards at each site and the trial was overseen by an independent data and safety monitoring committee. All participants provided written informed consent. No compensation was given for participation. Medical physicians at the military treatment facilities referred patients with low back pain to the trial and performed examination and screening prior to participant enrollment. Participants were included in the trial if they were an active-duty military member reporting low back pain and aged 18–50 years. Participants were excluded for non-musculoskeletal pain, contraindication to spinal manipulation, recent spine fracture, recent spine surgery, diagnosis of post-traumatic stress disorder, or radiculopathy requiring further evaluation or referral.

### Interventions

Both usual medical care and chiropractic care were delivered as usual in the military treatment facilities. Care was not dictated by trial procedures. Usual medical care included the treatment of LBP by military medical physicians and was provided to participants in both trial arms. Usual medical care interventions delivered during the trial included education and self-management strategies, medications, and referral to other providers such as physical therapists or to pain management clinics. Chiropractic care in the trial included 6 weeks of care with up to 12 visits with an on-site chiropractic clinician. A description of the component interventions and diagnoses of chiropractic care delivered to trial participants has been previously reported [21]. Common interventions of chiropractic care included treatment of the low back and surrounding area with spinal manipulation, therapeutic exercise, hot/cold packs, and electrical muscle stimulation [21].

### Outcomes

The outcomes for this secondary analysis follow the design of the trial and were specified in a pre-registered analysis plan [25]. The primary outcomes of interest are pain-related disability measured with the 0–24 Roland-Morris Disability Questionnaire (RMDQ) [26] and average low back pain intensity in the previous week measured with a 0–10 numerical rating scale (NRS) [27]. The secondary outcomes of interest are the Patient-Reported Outcomes Measurement Information System (PROMIS)-29 [28] v1.0 domains of pain interference, physical function, fatigue, sleep disturbance, and social role. Previous work reported a low frequency of PROMIS-29 anxiety and depression symptoms in this trial sample which led to a skewed distribution which did not fulfill regression assumptions [29, 30]. Therefore, we did not analyze PROMIS-29 anxiety and depression outcomes for this sample. Outcomes were collected through an online data capture system which was supplemented by computer assisted telephone interviews, if needed. RMDQ and NRS were collected at baseline and weeks 2, 4, 6, 12, 26, 40, and 52 and PROMIS-29 was collected at baseline and weeks 6, 12, 26, 40, and 52. PROMIS outcome domains of physical function and social role were transformed by taking 100-(T-Score) to make a higher score indicative of worse health across all PROMIS domains.

### Sample size

The trial was powered to evaluate meaningful differences in the primary outcomes of RMDQ and NRS at 3 sites, enrolling 250 participants each. Prior to data analysis, we used standard deviations and correlation coefficients from the full trial sample to conduct a power analysis for 3 outcomes to determine the effect sizes at which we would have at least 80% power to detect between-group differences at alpha = 0.05 in mixed-effects models for a 154-participant sample. RMDQ was estimated to be 2.5 points at week 12 and 2.6 points at week 52. Pain intensity was estimated to be 1.1 points at weeks 12 and 52. PROMIS pain interference estimated to be 4.2 points at week 12 and 3.9 points at week 52. Recommended effect magnitude classifications for between-group differences include 1–2 points (small) and 2–5 points (moderate) for RMDQ,^16^ 0.5-1.0 points (small) and 1.0–2.0 points (moderate) for NRS,^16^ and 2–6 T-Score points for meaningful change in PROMIS measures [31].

### Randomization and allocation

Participants were randomly allocated 1:1, stratified by site, to the intervention trial arms of usual medical care plus chiropractic care or usual medical care alone using a computer-generated, adaptive, minimization algorithm. The algorithm balanced trial arms on participant characteristics of baseline age, sex, LBP duration, and pain intensity (worst in past 24 h). Allocation was concealed to participants and study personnel until after screening and enrollment in the trial.

### Blinding

Blinding of participants and trial clinicians was not possible in the trial design. This analysis was not blinded.

### Statistical analysis

We used SAS v9.4 (SAS Institute Inc., Cary, NC) to model each outcome in separate linear mixed-effects regression models over baseline and all follow-up time points. We followed the intent-to-treat principle analyzing participant data as assigned. Models included terms to adjust estimates by the allocation minimization covariates of baseline sex, age, pain intensity, and LBP duration and we included terms in the model for time, group and site as fixed effects and repeated measures of participants as random effects. We compared models with and without the three-way interaction term time*group*site and found this impacted some model estimates and improved model fit. Therefore, we included the three-way interaction in our models and report model estimates overall and by site. The primary endpoints of interest were 12 and 52 weeks. We report adjusted between-group mean differences and 95% confidence intervals from the models. Between-group mean differences were calculated in mixed effects models by taking UMC group – UMC + CC group, making a positive value indicative of a better outcome in the UMC + CC group. We also provide graphics of outcomes over time by site.

We performed sensitivity analyses to examine the effects of missing outcome data on results. We generated 25 datasets with multiple imputation under the missing at random assumption with chained equations using the previously described linear mixed-effects regression models. We then combined the results of the imputations and compared these to estimates of models using all observed data. For several outcomes, missing data substantially changed the effect estimates. Therefore, we chose to use a weighting procedure to create a complete dataset for statistical analysis.

We used an inverse probability weighting method for missing longitudinal data [32–34]. We first modelled the probability of missing data for each outcome using logistic regression and a stepwise covariate selection process with a conservative alpha = 0.25 for entrance and removal. We included the model covariates of time, group, site, sex, age, worst pain intensity, and LBP duration and used the stepwise selection process to assess the baseline covariates of race, ethnicity, RMDQ, NRS, and PROMIS outcome domains. These baseline characteristic variables were chosen to be assessed based on expectation of relatedness to missingness. We used linear mixed-effects regression models weighted by the inverse of the probability of missingness to generate predicted values for missing outcomes. Missing outcomes were replaced with these predicted values to generate a dataset consisting of complete data for all outcomes at all time points.

## Results

### Participants and treatment visits

**Fig. 1:**
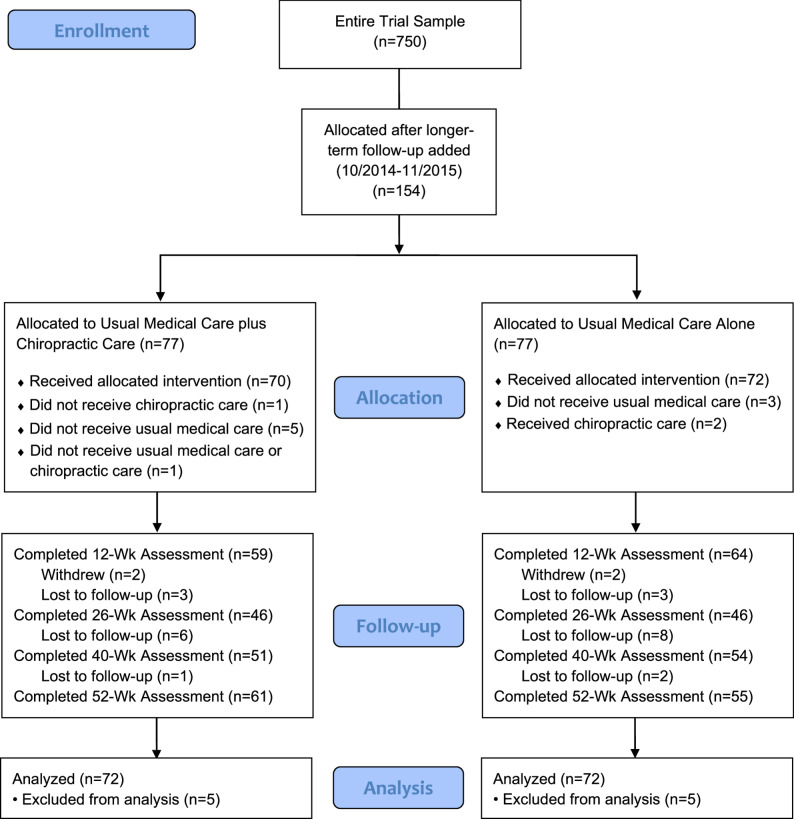
CONSORT flow diagram for participant inclusion

A flow diagram for the trial is shown in Fig. 1. Of the 750 total participants, 154 enrolled after the longer-term follow-up was added to the protocol in 9/2014. Missing data varied by follow-up time point. There was no missing baseline data and there were more missing data at later follow-up time points than at 6 and 12 weeks. At 12 weeks or later, non-missing outcome data for RMDQ and NRS ranged from 64 to 76% and from 42 to 86%. for PROMIS outcomes. In each trial arm, 2 participants withdrew and 3 were lost to follow-up before the 12-week follow-up and were excluded from this analysis. The result was an analysis of data from 144 participants.

Table 1 shows baseline participant data overall and by site for the 144 participants included in this analysis. Fifty-one of the participants were enrolled at the Pensacola site and 93 at Bethesda. The mean participant age was 33 years, 113 (78%) were male, 54 (38%) were non-white race, 18 (13%) were Hispanic or Latino ethnicity, and 71 (49%) had chronic LBP. However, baseline characteristics varied by site, with participants at the Pensacola site were younger with a higher percentage of male sex and white race. Participants reported moderate to high LBP-related disability, high pain interference, but less anxiety and depression symptoms than the general population (mean T-Scores < 50) and fatigue symptoms approximately equivalent to the general population (mean T-Score ≈ 50). Baseline measures were similar for most variables between trial arms and were consistent with baseline characteristics reported for the full trial sample [23].

**Table 1:** Baseline characteristics of participants receiving usual medical care plus chiropractic care vs. usual medical care alone by study site and overall (*n* = 144)

	Bethesda		Pensacola		Overall	
	UMC + CC (*n* = 47)	UMC (*n* = 46)	UMC + CC (*n* = 25)	UMC (*n* = 26)	UMC + CC (*n* = 72)	UMC (*n* = 72)
Age, years, mean (range)	35.1 (20–49)	34.1 (21–50)	28.1 (18–44)	30.1 (18–50)	32.7 (18–49)	32.7 (18–50)
Male, n (%)	34 (72)	34 (74)	23 (92)	22 (85)	57 (79)	56 (78)
Race, n (%)						
White	23 (49)	23 (50)	21 (84)	23 (88)	44 (61)	46 (64)
Black or African American	12 (26)	16 (35)	4 (16)	2 (8)	16 (22)	18 (25)
Other/Unspecified	12 (26)	7 (15)	0 (0)	1 (4)	12 (17)	8 (11)
Hispanic or Latino	5 (11)	6 (13)	2 (8)	7 (27)	7 (10)	13 (18)
LBP duration, n (%)						
Acute (< 3 months)	22 (47)	19 (41)	16 (64)	16 (62)	38 (53)	35 (49)
Chronic (≥ 3 months)	25 (53)	27 (59)	9 (36)	10 (38)	34 (47)	37 (51)
RMDQ, 0–24, mean (SD)	9.1 (5.8)	10.0 (5.9)	10.0 (6.5)	11.2 (5.1)	9.4 (6.0)	10.4 (5.7)
Pain intensity, 0–10, mean (SD)	3.9 (1.5)	4.1 (1.8)	4.8 (1.9)	4.8 (2.1)	4.2 (1.7)	4.3 (1.9)
PROMIS, T-Score, mean (SD)						
Pain Interference	59.1 (7.8)	58.8 (7.6)	61.7 (7.2)	61.2 (7.1)	60.0 (7.6)	59.7 (7.5)
Physical Function*	55.2 (8.0)	55.8 (6.4)	58.9 (5.4)	58.4 (6.1)	56.5 (7.4)	56.8 (6.4)
Sleep Disturbance	55.6 (7.2)	53.0 (7.1)	54.7 (6.2)	55.3 (10.3)	55.3 (6.9)	53.8 (8.4)
Fatigue	51.2 (10.2)	50.8 (9.5)	52.8 (10.3)	50.7 (12.8)	51.7 (10.2)	50.7 (10.7)
Anxiety	45.6 (6.9)	44.8 (7.0)	47.2 (7.4)	48.8 (10.6)	46.2 (7.0)	46.2 (8.6)
Depression	44.5 (5.6)	42.4 (3.8)	44.2 (5.5)	45.3 (8.8)	44.4 (5.5)	43.4 (6.2)
Social Role*	54.0 (9.0)	53.6 (8.7)	53.4 (6.9)	57.6 (9.8)	53.8 (8.3)	55.1 (9.3)

*UMC+CC* Usual Medical Care plus Chiropractic Care, *UMC* Usual Medical Care Alone, *RMDQ* Roland-Morris Disability Questionnaire

*PROMIS physical function and social role are transformed by 100-(T-Score), making a higher value indicative of worse health

### Outcomes

**Fig. 2:**
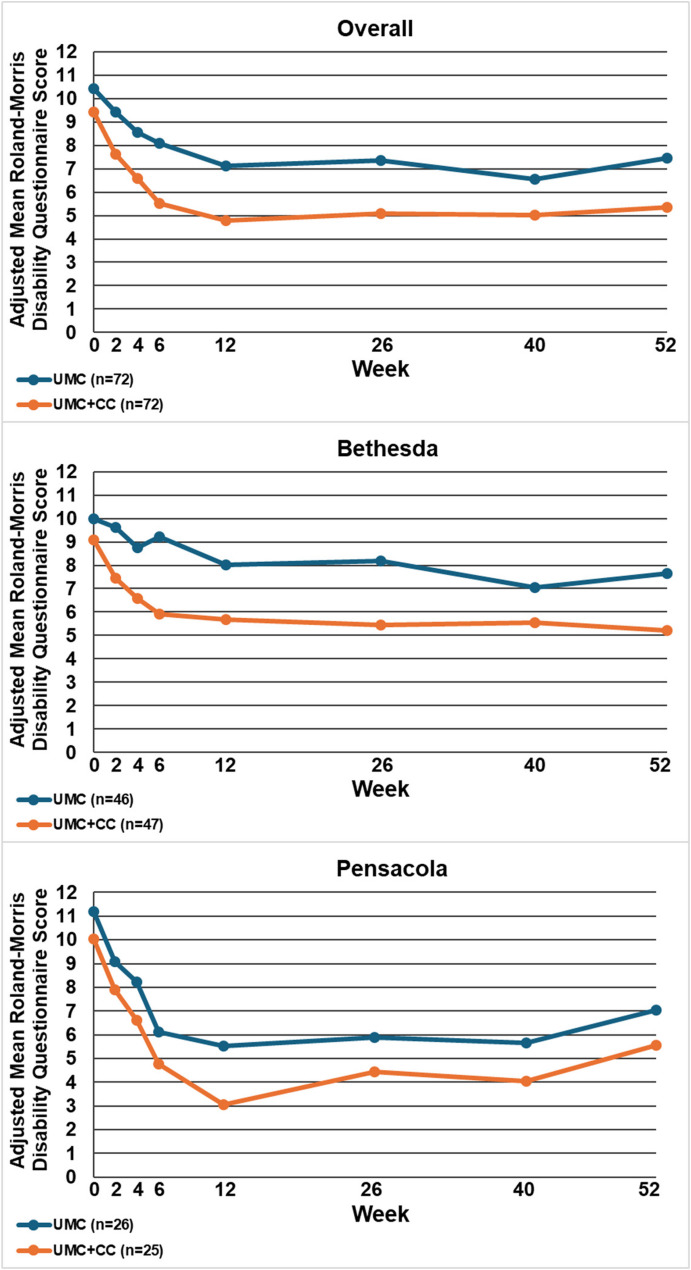
Within-group mean for Roland-Morris Disability Questionnaire (0-24) overall and by site at each follow-up time point. UMC: Usual Medical Care Alone; UMC+CC Usual Medical Care plus Chiropractic Care

**Fig. 3:**
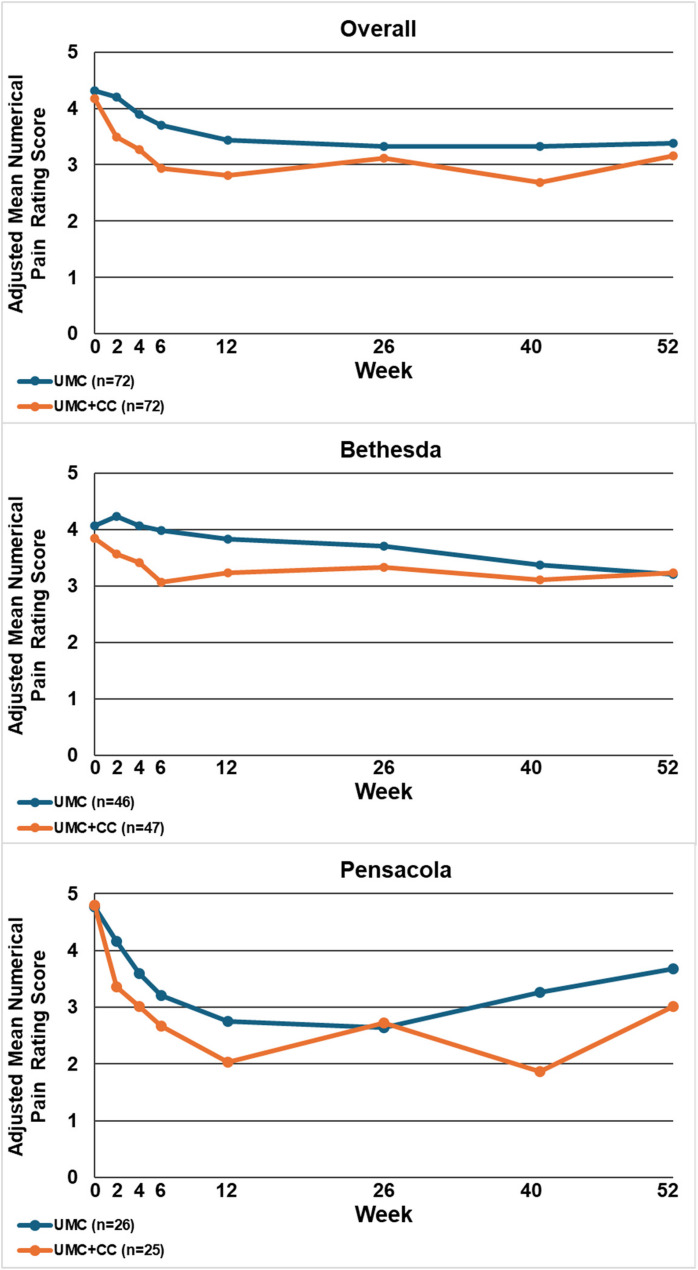
Within-group mean for average pain intensity within the last week measured with a numerical pain rating score (0-10) overall and by site at each follow-up time point. UMC: Usual Medical Care Alone; UMC+CC Usual Medical Care plus Chiropractic Care

**Fig. 4:**
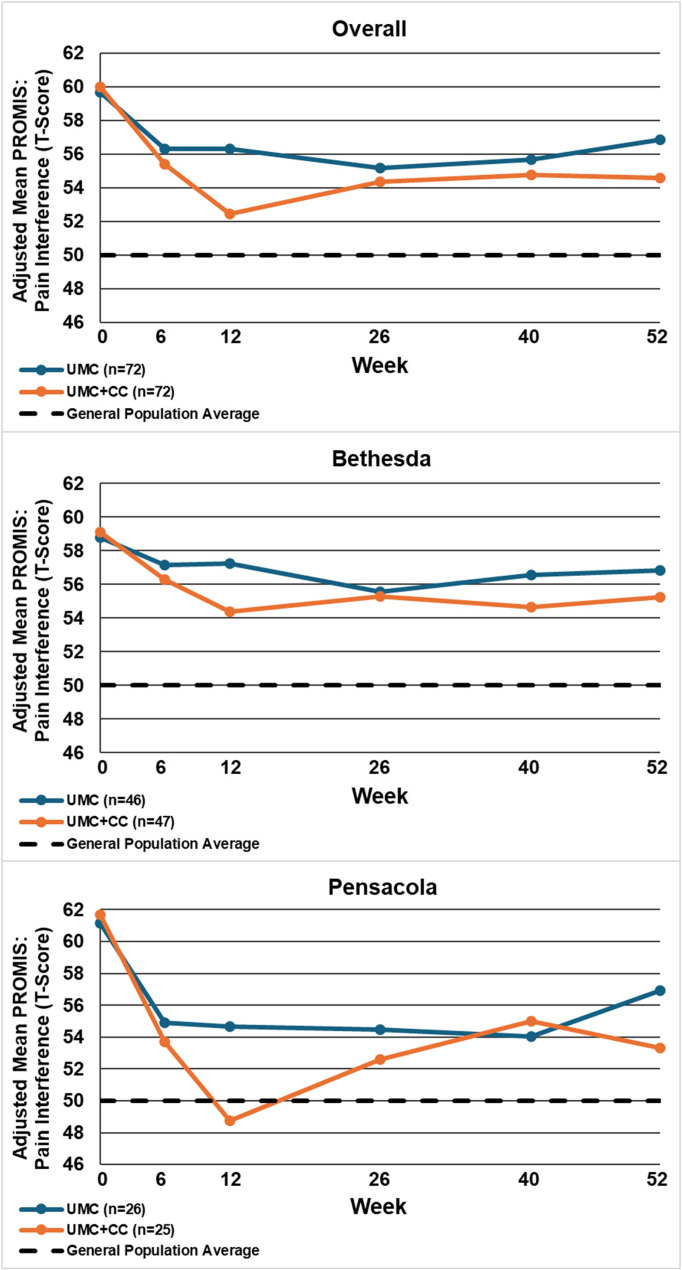
Within-group mean for PROMIS: pain interference (T-Score) overall and by site at each follow-up time point. UMC: Usual Medical Care Alone; UMC+CC Usual Medical Care plus Chiropractic Care

**Fig. 5:**
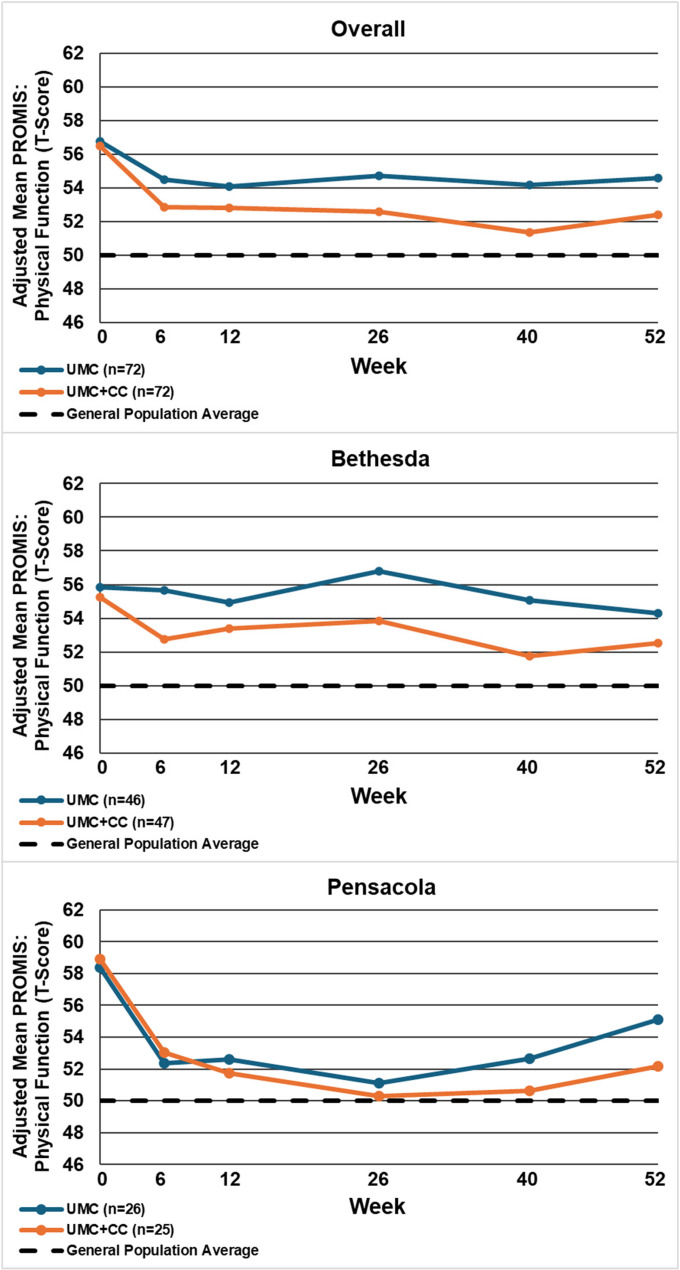
Within-group mean for transformed PROMIS: physical function (100-(T-Score)) overall and by site at each follow-up time point. UMC: Usual Medical Care Alone; UMC+CC Usual Medical Care plus Chiropractic Care

**Fig. 6:**
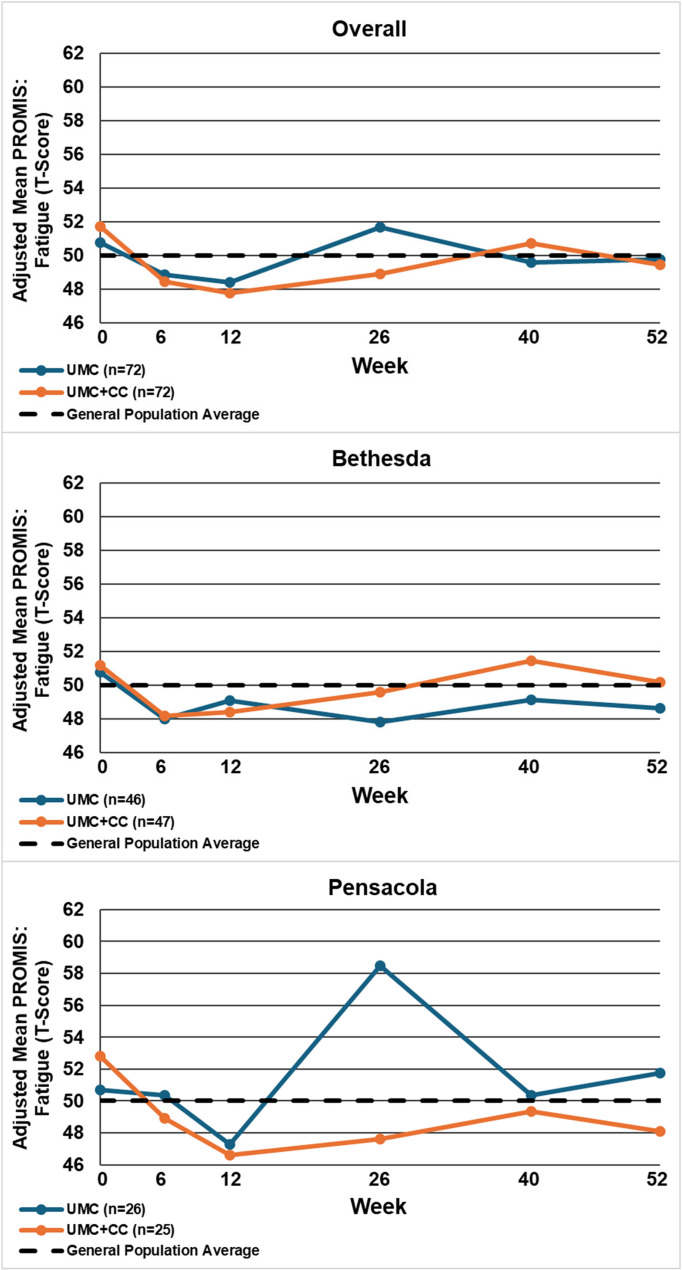
Within-group mean for PROMIS: fatigue (T-Score) overall and by site at each follow-up time point. UMC: Usual Medical Care Alone; UMC+CC Usual Medical Care plus Chiropractic Care

**Fig. 7:**
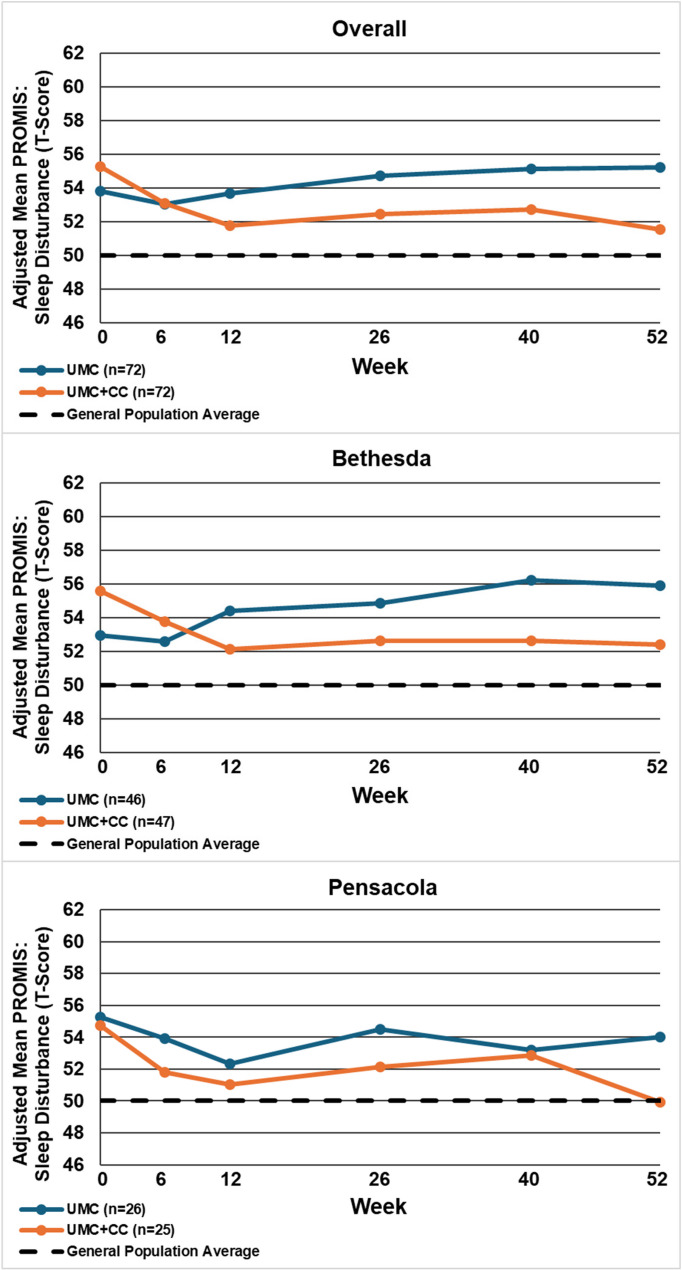
Within-group mean for PROMIS: sleep disturbance (T-Score) overall and by site at each follow-up time point. UMC: Usual Medical Care Alone; UMC+CC Usual Medical Care plus Chiropractic Care

**Fig. 8:**
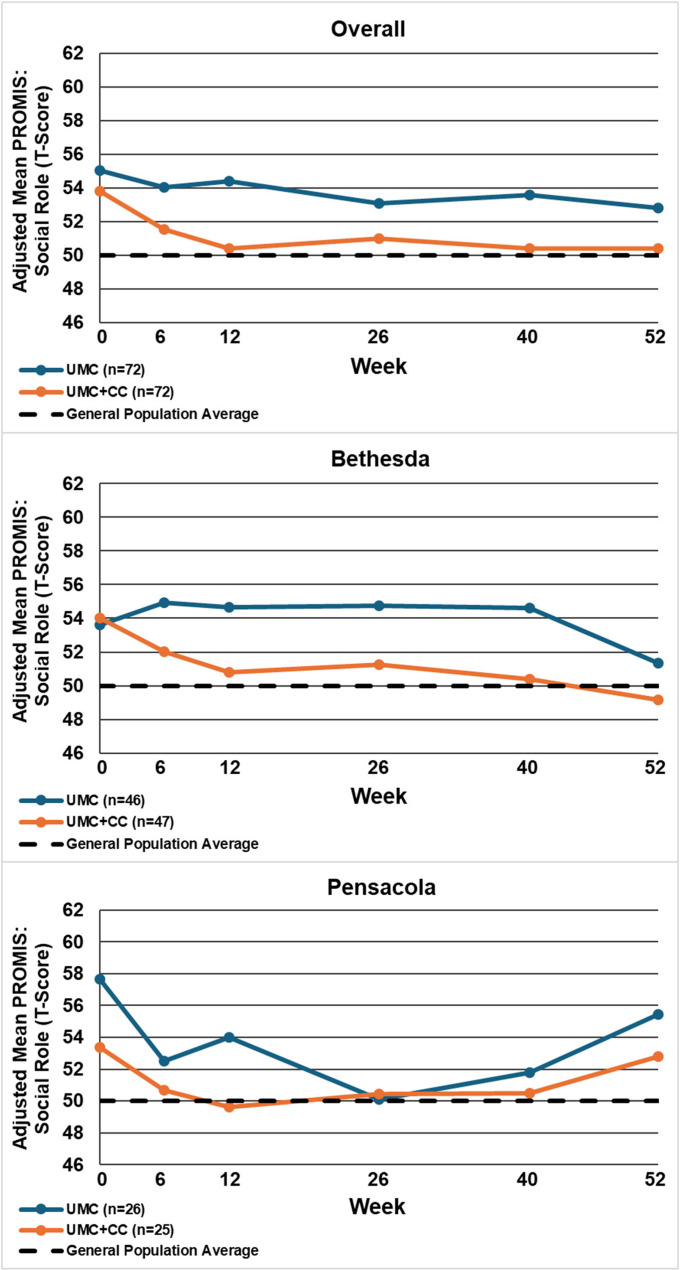
Within-group mean for transformed PROMIS: satisfaction with participation in social role (100-(T-Score)) overall and by site at each follow-up time point. UMC: Usual Medical Care Alone; UMC+CC Usual Medical Care plus Chiropractic Care

Table 2 displays the between-group mean differences and 95% confidence intervals for each outcome by site and overall, at 12 and 52 weeks. Figures 2, 3, 4, 5, 6, 7 and 8 show the estimated means at each outcome by site across all time points. Overall, between-group mean difference in RMDQ favored the UMC + CC group at weeks 12 and 52. The overall difference in NRS at week 12 was small in magnitude and diminished to a negligible difference at week 52. Overall, 12-week mean differences were noted for PROMIS pain interference (4.0 points, 95% CI 1.6 to 6.5) and social role (3.8 points, 95% CI 0.9 to 6.7). Compared to 12 weeks, mean differences at 52 weeks were smaller for pain interference (2.3 points, 95% CI -0.7 to 5.2) and social role (2.1 points, 95% CI -0.5 to 4.7), but were larger for physical function (2.1 points, 95% CI -0.8 to 4.9) and sleep disturbance (3.6 points, 95% CI 0.5 to 6.7).

**Table 2:** Between-group mean differences and 95% confidence intervals by site and overall at the primary endpoints of weeks 12 and 52

Outcome		Bethesda (*n* = 93)	Pensacola (*n* = 51)	Overall (*n* = 144)
	Week	Mean Difference	Mean Difference	Mean Difference
RMDQ	12	2.2 (0.04 to 4.3)	2.1 (-0.8 to 5.0)	2.2 (0.4 to 4.0)
	52	2.3 (0.02 to 4.6)	1.2 (-1.9 to 4.3)	1.7 (-0.2 to 3.7)
NRS	12	0.5 (-0.2 to 1.3)	0.6 (-0.4 to 1.6)	0.6 (-0.1 to 1.2)
	52	-0.1 (-1.0 to 0.8)	0.6 (-0.7 to 1.8)	0.2 (-0.5 to 1.0)
PROMIS: Pain Interference	12	2.6 (-0.3 to 5.5)	5.5 (1.5 to 9.4)	4.0 (1.6 to 6.5)
	52	1.3 (-2.1 to 4.8)	3.2 (-1.6 to 7.9)	2.3 (-0.7 to 5.2)
PROMIS: Physical Function	12	1.4 (-1.5 to 4.2)	0.5 (-3.3 to 4.3)	0.9 (-1.4 to 3.3)
	52	1.6 (-1.8 to 5.0)	2.6 (-2.0 to 7.1)	2.1 (-0.8 to 4.9)
PROMIS: Fatigue	12	0.5 (-3.6 to 4.6)	0.4 (-5.2 to 6.0)	0.4 (-3.1 to 3.9)
	52	-1.7 (-6.9 to 3.6)	3.4 (-3.7 to 10.5)	0.9 (-3.6 to 5.3)
PROMIS: Sleep Disturbance	12	2.1 (-1.3 to 5.4)	1.0 (-3.5 to 5.6)	1.5 (-1.3 to 4.4)
	52	3.3 (-0.4 to 7.0)	3.9 (-1.1 to 8.8)	3.6 (0.5 to 6.7)
PROMIS: Social Role	12	3.6 (0.1 to 7.1)	4.0 (-0.7 to 8.7)	3.8 (0.9 to 6.7)
	52	1.9 (-1.1 to 5.0)	2.3 (-1.9 to 6.4)	2.1 (-0.5 to 4.7)

A positive value favors the usual medical care plus chiropractic care group; Difference by site is difference in group*site*time interaction term; Overall difference is difference in group*time interaction term

*RMDQ* Roland Morris Disability Questionnaire, *NRS* Numerical pain rating scale

## Discussion

We found that adding chiropractic care to usual medical care resulted in moderate improvement in pain-related disability and small improvement in pain intensity in the short-term. These findings are consistent with those reported in the original analysis of 6 and 12 week outcomes from the entire 750 participant sample [23]. At 52 weeks, however, we found a small difference in pain-related disability and negligible difference in pain intensity. For PROMIS domains, differences favoring the addition of chiropractic care for pain interference and social role reduced over the course of longer-term follow-up. The greatest longer-term difference in PROMIS-29 domains was for sleep disturbance, which showed a meaningful difference at 52 weeks. Previous work from this trial also reported short-term improvement in sleep disturbance [24]. Our finding that this benefit was sustained at 52 weeks is potentially important given the established relationship between sleep quality and musculoskeletal pain in the military [35] and beyond [36–41]. Further study of the effect of chiropractic care on sleep quality is needed to determine if our findings are reproducible.

Active-duty military enrolled in the trial reported very few mental health symptoms at baseline. The mean PROMIS anxiety and depression T-Scores were better than the general population average as indicated by T-Scores < 50. While it is possible that a young and physically active population, characteristic of U.S. active-duty military, may experience less mental health symptoms, it is widely recognized that mental health symptoms are underreported by U.S. military members [42–44]. Future study of populations reporting mental health symptoms is needed to better understand longer-term effects of chiropractic care on mental health.

Chiropractic care delivered to trial participants included a number of nonpharmacological interventions, most commonly spinal manipulation and therapeutic exercise [21]. Compared to usual care, a combination of patient reassurance, recommendations on exercise and avoiding passive treatments, over the counter pain medication, and spinal manipulation delivered by chiropractors has led to greater improvement in functioning, but not pain [45]. In a more direct comparison to the population studied in our trial, previous work has shown that U.S. military members with LBP receiving early treatment with nonpharmacological approaches, including chiropractic care, achieve greater improvement in function one year after initiation of care compared to treatment without early nonpharmacological approaches [46].

Work comparing spinal manipulation to back school and individualized physiotherapy in an outpatient rehabilitation setting reported greater improvement in function and pain at 52 weeks with spinal manipulation, but with a substantially greater number of visits [47]. Interventions requiring a greater number of clinic visits may pose an implementation challenge for military treatment facilities, where U.S. military members have limited access to follow-up care for LBP [48]. As a consequence of limited access, care for U.S. military members with LBP is largely delivered outside of military treatment facilities through coverage provided by TRICARE. However, TRICARE does not cover chiropractic care [49]. A strength of our pragmatic trial design is that it evaluated outcomes with the addition of chiropractic care to usual medical care in military treatment facilities, the current mode through which U.S. military personnel receive covered chiropractic care.

This study provides additional evidence that care delivered by chiropractors is an effective approach for producing small to moderate effects on outcomes in the treatment of LBP. Results further suggest that active-duty military personnel receiving chiropractic care in military treatment facilities have improved quality of life outcomes in the short and longer term. Barriers to implementing these findings include a limited number of chiropractors employed in military treatment facilities and a lack of TRICARE coverage for chiropractic care in the private sector. Health policy changes are necessary to address barriers to chiropractic care access for delivery at scale.

### Limitations

This secondary analysis was not powered to detect small differences that are typical of nonpharmacological interventions for pain. The power calculation conducted prior to our analysis suggested the sample would provide power to detect moderate between-group differences for the outcomes. Our effect estimates for the 52-week outcomes of pain interference, physical function, and social role were just above 2 T-Score points, the lower threshold of suggested meaningful difference for PROMIS measures [31].

There was substantial missing outcome data in the longer-term follow-up due to this trial being conducted in a mobile active-duty military population. Rather than reporting only complete cases, we followed a process of assessing the effects of missing data on effect estimates. To address bias associated with missing outcome data, we used a conservative approach in selecting covariates and replacing missing outcome values before analyzing a complete dataset [50].

Health care utilization during the longer-term follow-up was not collected, limiting the ability to assess differences in care seeking over the longer-term. There was a possibility of cross-contamination over the course of follow-up, whereby participants could have received care that was beyond the scope of the intervention assigned in the trial. Possible contributors to site differences include structural barriers, e.g. access to care or resource availability constraining the number of healthcare visits, varying clinician approach, and/or varying patient characteristics, among others. Future research aimed at differentiating these factors could be used to inform a more optimal approach to chiropractic care.

## Conclusions

Adding chiropractic care to usual medical care for active-duty U.S. military members with low back pain resulted in a small difference in pain-related disability and a meaningful difference in sleep disturbance but not pain intensity at 52 weeks. Differences in pain interference, physical function, and social role estimates also favored the addition of chiropractic care over the longer-term follow-up.

## Data Availability

The dataset analyzed in the current study is available from Palmer College of Chiropractic, on reasonable request.
